# Intestinal-type of differentiation predicts favourable overall survival: confirmatory clinicopathological analysis of 198 periampullary adenocarcinomas of pancreatic, biliary, ampullary and duodenal origin

**DOI:** 10.1186/1471-2407-13-428

**Published:** 2013-09-22

**Authors:** Peter Bronsert, Ilona Kohler, Martin Werner, Frank Makowiec, Simon Kuesters, Jens Hoeppner, Ulrich Theodor Hopt, Tobias Keck, Dirk Bausch, Ulrich Friedrich Wellner

**Affiliations:** 1 Institute of Pathology, University of Freiburg, Breisacher Str. 115a, Freiburg 79106, Germany; 2 Clinic for General and Visceral Surgery, University of Freiburg, Hugstetter Str. 55, Freiburg 79106, Germany; 3 Clinic for Surgery, University of Schleswig-Holstein Campus Lübeck, Ratzeburger Allee 160, Lübeck 23582, Germany

## Abstract

**Background:**

Periampullary adenocarcinomas comprise pancreatic, distal bile duct, ampullary and duodenal adenocarcinoma. The epithelia of these anatomical structures share a common embryologic origin from the foregut. With steadily increasing numbers of pancreatoduodenectomies over the last decades, pathologists, surgeons and oncologists are more often confronted with the diagnosis of “other than pancreatic” periampullary cancers. The intestinal subtype of ampullary cancer has been shown to correlate with better prognosis.

**Methods:**

Histological subtype and immunohistochemical staining pattern for CK7, CK20 and CDX2 were assessed for n = 198 cases of pancreatic ductal, distal bile duct, ampullary and duodenal adenocarcinoma with clinical follow-up. Routine pathological parameters were included in survival analysis performed with SPSS 20.

**Results:**

In univariate analysis, intestinal subtype was associated with better survival in ampullary, pancreatic ductal and duodenal adenocarcinoma. The intestinal type of pancreatic ductal adenocarcinoma was not associated with intraductal papillary mucinous neoplasm and could not be reliably diagnosed by immunohistochemical staining pattern alone. Intestinal differentiation and lymph node ratio, but not tumor location were independent predictors of survival when all significant predictor variables from univariate analysis (grade, TNM stage, presence of precursor lesions, surgical margin status, perineural, vascular and lymphatic vessel invasion, CK7 and CDX2 staining pattern) were included in a Cox proportional hazards model.

**Conclusions:**

Intestinal type differentiation and lymph node ratio but not tumor location are independent prognostic factors in pooled analysis of periampullary adenocarcinomas. We conclude that differentiation is more important than tumor location for prognostic stratification in periampullary adenocarcinomas.

## Background

The present WHO classification of tumors distinguishes between pancreatic ductal (PDAC), extrahepatic (distal) bile duct (DBDAC), ampullary (AMPAC) and small intestinal (including duodenal, DUOAC) adenocarcinoma [1]. A fundamental observation is that survival after resection of adenocarcinoma of periampullary location (pancreatic head, distal bile duct, ampulla, duodenum) differs greatly, with DUOAC and AMPAC displaying a much better survival than pancreatic head PDAC or DBDAC, implying several issues of continued debate [2-4].

First, due to the anatomical complexity of the periampullary region, correct classification with respect to location remains challenging to the pathologist. Usually the origin of a periampullary tumor is defined macroscopically by location of the main tumor mass or eventual precursor lesions and has to be confirmed microscopically [2-4]. There is still considerable debate on how localization of adenocarcinomas and their precursor lesions arising in this region should be assessed [2,5].

Another aspect is the question of the biological basis of the observed differences in survival. A major step was the recognition of the intestinal (INT) versus pancreatobiliary (PB) histopathologic phenotypes of AMPAC by Kimura et al. in 2004 [6]. The INT type proved to be associated with considerably better prognosis than the PB subtype, which has been confirmed by several recent series [3,4,7].

Our study aimed at a detailed analysis of clinical, pathological and immunohistochemical parameters for assessment of tumor biology and identification of prognostic factors after resection of periampullary adenocarcinomas of all four locations.

## Methods

### Patients and data

For the purpose of this study, periampullary adenocarcinomas were defined as pancreatic head PDAC, DBDAC, AMPAC or DUOAC. Only cases with resection by pancreatoduodenectomy, including conversion to total pancreatectomy due to positive intraoperative pancreatic resection margin were included. Thereby cases of the following WHO tumors [1] were excluded: PDAC not located in the pancreatic head, solid-pseudopapillary, acinar and neuroendocrine neoplasms, benign lesions, pancreatoblastoma, teratoma, mesenchymal tumors, lymphoma and secondary tumors. Patients operated at the Clinic for General and Visceral Surgery, University of Freiburg from 2001 to 2011 were identified and baseline and follow-up data extracted from a prospectively maintained database. All histopathological workup was performed at the Insitute of Pathology, University of Freiburg. Archived hematoxylin & eosin (H&E) stained slides were reevaluated by two experienced pathologists (PB, IK) for accuracy of diagnosis and formalin-fixed and paraffin-embedded tissue (FFPE) blocks were selected for generation of serial tissue slices for immunohistochemistry (IHC). All cases with sufficient available FFPE for IHC were included in the study. The study protocol was approved by the Ethics Committee of the University of Freiburg (Ref 13/11).

### Standard pathological assessment

During the study period (2001–2011), a standardized protocol was followed for diagnostic workup of pancreatoduodenectomy specimens: First, resection margins including the closest margin to the tumor and retroperitoneum towards portal vein and superior mesenteric artery were marked intraoperatively by the surgeon. After intraoperative transfer to the institute of pathology, every specimen was examined macroscopically by an experienced pathologist. Identifiable tumor masses or suspect areas were measured in three dimensions. Localization, size and distance of the tumor to the resection margins were documented and lymph node stations were separately evaluated. After formalin fixation and paraffin embedding, tissue slices of 3 μm thickness were H&E stained. The following routine work up was equal in procedure for pylorus preserving pancreatoduodenectomy and the classical Whipple procedure. The number of routine tissue blocks and corresponding tissue slices was at minimum 10 (range 10 to 17). The standardized protocol comprised at least two samples for the enteral (oral and aboral) resection margins, and one sample for each of the following locations: whole circumferential parenchymal pancreas resection margin, tumor in relation to the closest posterior (retroperitoneum and vascular groove) resection margin, resection margin at the common bile duct, tumor in relation to the common bile duct and the main duct of the pancreas, tumor in relation to the duodenum, Papilla vateri. At minimum twelve regional lymph nodes were embedded in at least two routine tissue blocks. In case of portal venous en-bloc resections, one additional tissue sample in relation to the tumor was embedded. Additional tissue biopsies were embedded upon request of the operating surgeon. This standardized protocol was modified for total pancreatectomy as follows. The number of routine tissue blocks and corresponding tissue slices was minimal 11 (range 11 to 19). The resection margin of the splenic artery and vein and one sample of the spleen were embedded additionally, while the whole circumferential parenchymal pancreas resection margin was not embedded.

Histopathological reports included diagnosis according to WHO classification, UICC stage, presence or absence of lymphatic, vascular and perineural invasion and assessment of oral, aboral, biliary and posterior (retroperitoneal and vascular groove) resection margin. Additional immunohistochemistry for pancytokeratin was performed when it was felt necessary in difficult cases.

### Histologic workup

**Figure 1:**
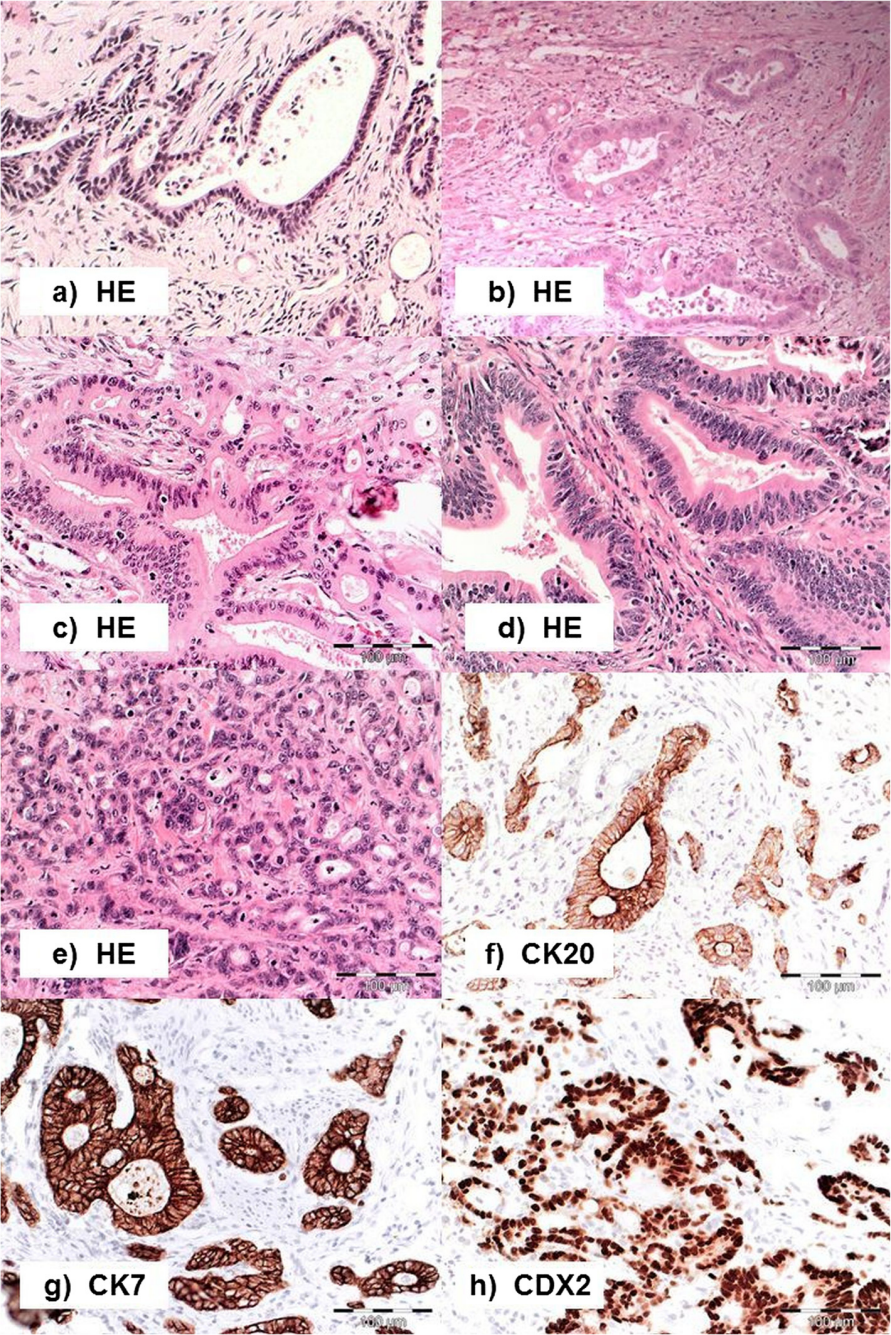
**Histopathological Subtypes of Periampullary Adenocarcinomas. (a-e)** hematoxylin-eosin stained tissue (H&E): **(a, b)** adenocarcinoma of the pancreatobiliary type with simple branching glands lined by cuboidal columnar tumor cells with rounded nuclei and focally cribriform growth pattern. **c** adenocarcinoma of the mixed type showing both intestinal and pancreatobiliary growth pattern evenly distributed, **d** adenocarcinoma of the intestinal type with characteristic branching tubular glands, simple and pseudostratified mucin producing glandular epithelium with elongated hyperchromatic and pseudostratified nuclei, **(e)** poorly differentiated carcinoma displaying solid insular glandular growth pattern and remarkable nuclear atypia; **(f-h)** typical immunohistochemical staining patterns for cytokeratin 7 (CK7) in a pancreatobiliary type adenocarcinoma **(g)**, cytokeratin 20 (CK20) **(f)** and caudal type homeobox 2 (CDX2) **(h)** in an intestinal type adenocarcinoma, pictures taken at 20-fold magnification.

For the present study, all cases were re-assessed by a surgeon (UFW) and two experienced pathologists (PB, IK) in terms of clinical findings and history, preexisting macro- and histopathologic reports and H&E stained tissue slides, as well as new H&E stained tissue slices to ensure correct diagnosis according to current WHO classification and anatomical tumor location. The site of the main tumor mass and/or eventual precursor lesions (intraductal papillary mucinous neoplasms of the pancreas (IPMN) or adenomatous lesions of duodenum, ampulla or bile duct) was used for definition. For this study, five histological subtypes were defined. Intestinal (INT), pancreatobiliary (PB) and mixed intestinal-pancreatobiliary (MIX), as well as poorly differentiated (POOR) were defined according to Albores-Saavedra et al. [8]. An additional category was added for rare other phenotypes (OTH), as mucinous and adenosquamous adenocarcinomas were found in our collective. According to Albores-Saavedra et al. [8], INT adenocarcinomas are characterized by well-formed tubular to elongate glands, complex cribriformed areas, and solid nests similar to colorectal adenocarcinoma, whereas PB adenocarcinomas show simple or branching glands and small solid nests of cells surrounded by abnormal desmoplastic stroma (Figure 1). Few tumors with both patterns equally distributed were assigned to the MIX type. Poorly differentiated carcinomas (POOR) are composed of solid sheets and nests admixed with densely packed, small irregular glands and individual cells with marked nuclear polymorphism, little or no mucin production and strong mitotic activity (Figure 1).

Tumors containing over 50% of stromal mucin were classified as mucinous, as proposed by the WHO classification [1] of ampullary tumors, while diagnosis of colloid carcinoma of the pancreas requires at least 80% of extracellular mucin pools combined with a characteristic well-differentiated cuboidal to columnar cellular morphology.

Histopathologic assessment was done by two independent pathologists blinded for the respective clinical outcome. For classification of histopathologic subtype and immunohistochemistry, the pathologists were blinded towards tumor location.

### Immunohistochemistry

After individual case review, tissue slides of 3 μm were prepared from representative FFPE blocks. Immunohistochemistry was carried out using commercially available ready-to-use antibodies for cytokeratin 7 (CK7, DAKO IR619), cytokeratin 20 (CK20, DAKO IR777), and caudal type homeobox 2 (CDX2, DAKO IR080), LINKER reagent (DAKO K8022) and EnVision™ Flex Visualization system (DAKO K8000) on an autostainer LINK 48 (DAKO, Hamburg, Germany) device according to the manufacturer's instructions. The specimens were counterstained with hematoxylin. Omission of primary antibodies served as negative controls and normal pancreatic and intestinal epithelia as internal positive control.

Representative images of immunohistochemical stains are shown in Figure 1. Immunostaining for CK7 and CK20 was considered positive when appropriate brown staining was seen in the tumor cell cytoplasm and immunostaining for CDX2 when appropriate brown staining was seen in the nucleus. CK7, CK20 and CDX2 expressions were established calculating the percentage of immunoreactive cells in the total number of tumor cells and rounding to decades. Only cases with more than 5% of positive tumor cells were regarded as positive.

### Statistics

IBM SPSS Statistics Version 20 (SPSS Inc. Chicago, IL) was used for all statistical calculations. For exploratory and descriptive analysis, scale variables were expressed as median (range) and ordinal or dichotomous variables as absolute and relative frequencies. For assessment of diagnostic consistency, interrater reliability analysis using the Kappa statistic was performed. Survival data was plotted and analyzed according to the Kaplan-Meyer method. For univariate and multivariate statistical testing the following methods were used: Mann–Whitney test, Chi squared test, Spearman rank correlation, binary logistic regression, Logrank test and Cox regression. The significance level was set to p = 0.05 and all statistical tests were performed two-sided.

## Results

### Clinico-pathological reassessment and baseline data

**Figure 2:**
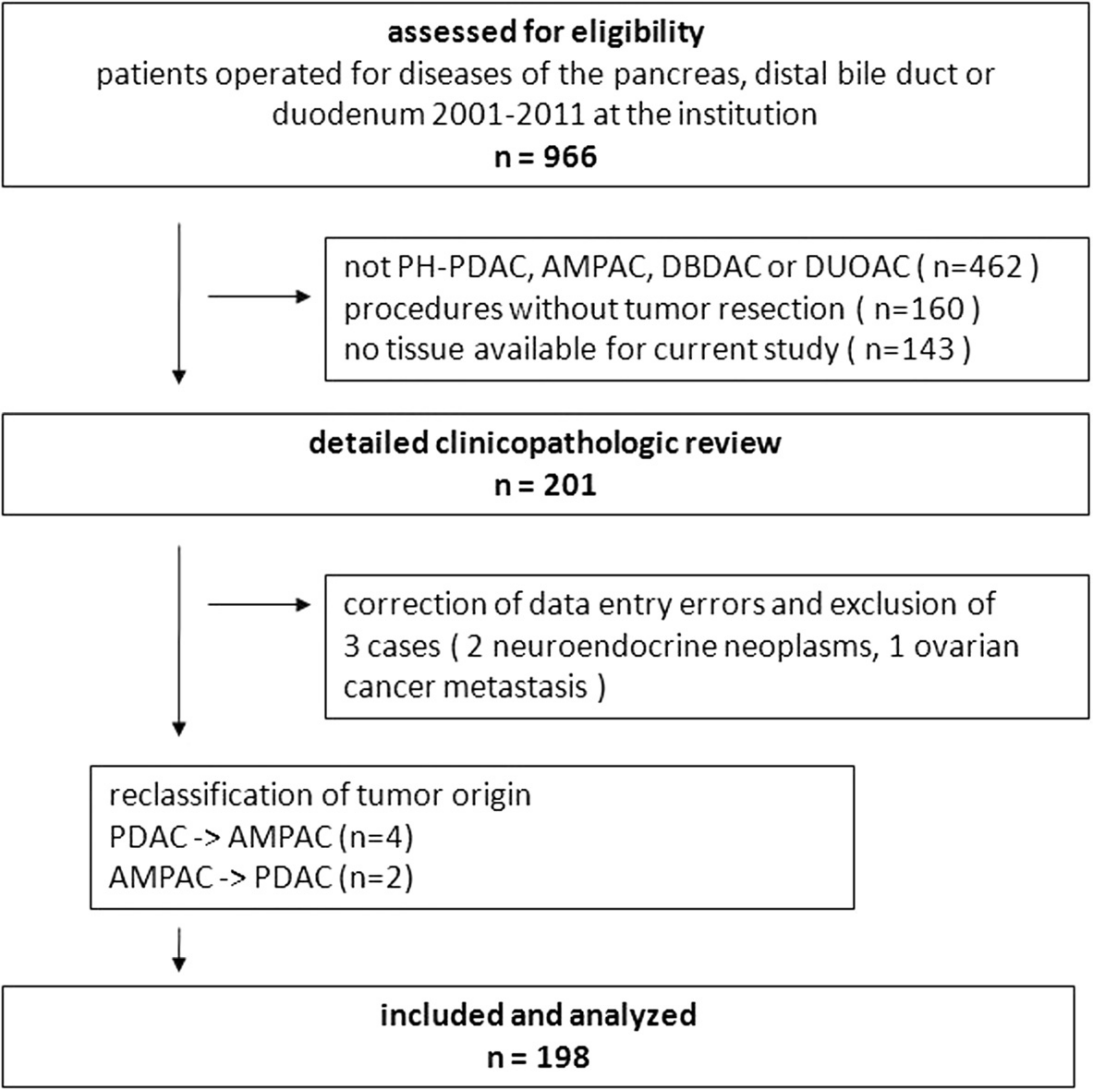
**Flowchart of patient selection and data assessment.** Abbreviations: PH-PDAC pancreatic head pancreatic ductal adenocarcinoma, DBDAC distal bile duct adenocarcinoma, AMPAC ampullary adenocarcinoma, DUOAC duodenal adenocarcinoma.

From a total of 966 surgical patients assessed for eligibility (Figure 2), n = 462 did not have periampullary adenocarcinomas, n = 160 had no tumor resection and in n = 143 cases there was insufficient tissue left for the present study due to use for other studies. Reassessment of clinical and pathological reports as well as histopathologic reexamination of H&E stained tissue slides in 201 cases resulted in correction of data entry errors and thus exclusion of two patients with neuroendocrine neoplasms and one patient with ovarian cancer metastasis to the periampullary region. Furthermore there was a change in diagnosis from PDAC to AMPAC in four patients and from AMPAC to PDAC in two patients. Follow-up survival data was complete due to regular follow-up within the Comprehensive Cancer Center Freiburg. Out of the 198 patients included in the study, about two third had pancreatic cancer (n = 126). The second largest group was ampullary cancer (n = 40), followed by distal bile duct (n = 23) and duodenal cancer (n = 9) (Table 1).

**Table 1:** Baseline parameters, basic pathology and immunohistochemical markers for 198 patients with periampullary adenocarcinomas

**Parameter**		**Total**	**PDAC**	**DBDAC**	**AMPAC**	**DUOAC**	**p**
**N**		**198**	**126**	**23**	**40**	**9**	**-**
Male : female		102:96	63:63	14:9	22:18	3:6	0.511
Age		67 (30–89)	67 (30–89)	69 (49–83)	65 (36–84)	66 (46–78)	0.137
Distant metastasis (M1)		7 / 4%	4 / 3%	0 / 0%	2 / 5%	1 / 11%	0.448
Neoadjuvant therapy		14 / 7%	13 / 10%	0 / 0%	1 / 3%	0 / 0%	0.126
Resection	PPPD	163	103	19	34	7	0.803
	Whipple	23	14	2	5	2	
	total PE	12	9	2	1	0	
	PVR	62 / 31%	53 /42%	6 / 26%	2 / 5%	1 / 11%	**0.000**
Perioperative mortality		7 / 4%	5 / 4%	2 / 9%	0 / 0%	0 / 0%	0.300
Tumor size (mm)		25 (1–320)	27 (1–80)	15 (8–25)	20 (2–320)	40 (22–60)	**0.000**
Grade G3/4		72 / 37%	48 / 39%	8 / 35%	12 / 31%	4 / 44%	0.785
Stage T3/4		155 / 78%	109 / 87%	18 / 78%	21 / 53%	7 / 78%	**0.000**
Stage N1/2		132 / 67%	89 / 71%	14 / 61%	24 / 60%	5 / 56%	0.468
Lymph node ratio (LNR)		.09 (0 - .86)	.10 (0 - .86)	.08 (0 - .72)	.07 (0 - .71)	.10 (0 - .28)	0.694
Lymphangiosis (L1)		87 / 44%	60 / 48%	5 / 22%	19 / 48%	3 / 33%	0.116
Hemangiosis (V1)		29 / 15%	22 / 18%	3 / 13%	2 / 5%	2 / 22%	0.237
Perineural invasion (Pn1)		113 / 57%	88 / 70%	12 / 52%	13 / 33%	0 / 0%	**0.000**
Associated precursor	Adenoma	20 / 10%	1 / 1%	4 / 17%	12 / 30%	3 / 33%	**0.000**
	IPMN	11 / 6%	11 / 9%	0 / 0%	0 / 0%	0 / 0%	0.084
Positive resection margin		51 / 26%	41 / 33%	5 / 22%	3 / 8%	2 / 22%	**0.017**
**Subtype**							
INT		39 / 20%	11 / 9%	5 / 22%	18 / 45%	5 / 56%	**0.000**
MIX		12 / 6%	8 / 6%	2 / 9%	2 / 5%	0 / 0%	
PB		118 / 16%	89 / 71%	12 / 52%	15 / 38%	2 / 22%	
OTH		6 / 3%	5 / 4%	0 / 0%	0 / 0%	1 / 11%	
POOR		23 / 12%	13 / 10%	4 / 17%	5 / 13%	1 / 11%	
**Immunohistochemical Markers**							
% CK7+		90 (0–100)	90 (0–100)	95 (0–100)	80 (0–100)	0 (0–60)	**0.000**
% CK20+		0 (0–100)	0 (0–100)	0 (0–100)	10 (0–100)	70 (10–100)	**0.000**
% nuclear CDX2+		10 (0–100)	0 (0–100)	0 (0–100)	50 (0–100)	95 (60–100)	**0.000**

Values are depicted as absolute and percentage of column for categorial and median (range) for scale variables if not otherwise specified. p value derived from two-sided Median test (scale variables), Chi squared test (categorial variables) and from two-sided Spearman rank correlation test (immunohistochemical markers), each for the respective rows.

Abbreviations: *PPPD* pylorus preserving pancreatoduodenectomy, Whipple classical Whipple procedure, *PE* pancreatectomy, *PVR* portal venous resection. *INT* intestinal, *PB* pancreatobiliary, *MIX* mixed intestinal-pancreatobiliary, *POOR* poorly differentiated carcinoma, *OTH* other specific subtypes, + positive, *CK* cytokeratin, *PDAC* pancreatic ductal adenocarcinoma, *DBDAC* distal bile duct adenocarcinoma, *AMPAC* ampullary adenocarcinoma, *DUOAC* duodenal adenocarcinoma.

Clinical and baseline data are shown in Table 1. No significant differences were found concerning age or gender distribution among the different tumor locations. 14 patients (7%) had received neoadjuvant therapy before resection (13 PDAC and 1 AMPAC). Distant metastasis was very rare among resected patients (4%). The usual resection procedure was pylorus-preserving pancreatoduodenectomy (PPPD, n = 163), with few cases requiring extension to classical Whipple procedure (CWP, n = 23) or total pancreatectomy (TPE, n = 12) for tumor invasion of the pyloric region or intraoperative positive pancreatic resection margin, respectively. PDAC and DBDAC were significantly more often locally advanced as demonstrated by a higher rate of portal venous resections (PVR) due to adhesion to or infiltration of the portal vein or it’s confluence (PDAC/DBDAC 42%/26% vs 5%/11% AMPAC/DUOAC). Perioperative mortality was 4% (n = 7).

### Basic pathology and histological subtype analysis

Results of explorative data analysis and statistical testing concerning basic pathology are depicted in Table 1. Several statistically significant differences between the four groups could be noted. DUOAC had the largest median diameter (40 mm) while AMPAC (20 mm) and DBDAC (15 mm) were smaller and PDAC intermediate (27 mm). Only 53% of AMPAC were of stage T3 or T4, compared to 78-87% for the other entities. Likewise, only 8% of AMPAC had positive resection margins, compared to 22-33% for other locations. Adenomatous precursor lesions displayed statistically significant decreasing frequency from DUOAC/AMPAC (33%/30%) to DBDAC/PDAC (17%/1%). Nine percent of PDAC were associated with IPMN precursor lesions. High-grade intraepithelial dysplasia was present in 17 of 20 (85%) associated adenomatous precursor lesions and in 9 of 11 (82%) IPMN.

Results of histological subtype assessment are shown in Table 1. The distribution of subtypes was significantly different among the adenocarcinoma groups. The INT type adenocarcinoma showed decreasing frequency with distance from the duodenum in the order DUOAC – AMPAC - DBDAC - PDAC, ranging from 56% to 9%, while the percentage of PB type increased from 22% to 71%. Of note, two DUOAC were assigned to the PB phenotype by application of the subtype criteria. MIX and OTH types were not frequently encountered (0 - 9% and 0 – 11%). The percentage of poorly differentiated carcinomas was relatively homogenous ranging from 10 to 17%. Rare WHO subtypes designated as OTH were mucinous (2 PDAC and 1 DUOAC) and adenosquamous (3 PDAC). Interrater reliability concerning the five defined histological subtypes was found to be very good (Kappa = 0.920, p = 0.000).

### Diagnostic value of immunohistochemical markers

Results of immunohistochemical marker assessment are presented in Table 1. Cytokeratin 7 (CK7) is a marker of pancreatobiliary epithelia, whereas cytokeratin 20 (CK20) and CDX2 are expressed by intestinal epithelia. Median intestinal marker expression was highest in DUOAC (CK20 and CDX2, 70% and 95%) and decreased for AMPAC (10% and 50%) and negative for DBDAC and PDAC (median expression level 0%). The pancreatobiliary marker CK7 showed the inverse pattern with high median expression level in PDAC / DBDAC (90%/ 95%), decreased with AMPAC (85%) and 0% in DUOAC. These correlations were highly significant (p < 0.001 in two-sided Spearman rank correlation test).

Uni- and multivariate binary logistic regression models were used in the attempt to predict the INT type by means of the immunohistochemical markers CK7, CK20 and CDX2 (details shown in Table 2). However, due to poor performance for prediction of the INT type in PDAC / DBDAC location (0%-13% correct prediction for univariate and 19% for multivariate model), even a multivariate model including all three markers only achieved 51% correct prediction for the INT type when applied to the total patient collective.

**Table 2:** Diagnostic value of immunohistochemical markers for intestinal type adenocarcinoma

**Location**	**Marker**	**% Positive tumor cells**		**% Correct prediction**		**p**
		**Median (Range)**				
		**INT**	**NON-INT**	**INT**	**NON-INT**	
PDAC / DBDAC	CK7	90 (0–100)	90 (0–100)	0%	100%	0.288
	CK20	10 (0–100)	0 (0–100)	0%	100%	0.034
	CDX2	45 (0–100)	0 (0–90)	13%	100%	0.000
	CK7, CK20, CDX2	-		**19%**	**100%**	**0.002**
	multivariate					
AMPAC / DUOAC	CK7	20 (0–100)	85 (0–100)	57%	75%	0.024
	CK20	80 (0–100)	8 (0–100)	65%	79%	0.002
	CDX2	90 (20–100)	30 (0–100)	87%	79%	0.000
	CK7, CK20, CDX2	-		**87%**	**75%**	**0.000**
	multivariate					
ALL	CK7, CK20, CDX2	-		**51%**	**95%**	**0.000**
	multivariate					

Prediction modeled by univariate and multivariate binary logistic regression analysis, p values given for the two-sided overall model omnibus test of the respective row.

Abbreviations: *PDAC* / *DBDAC* / *AMPAC* / *DUOAC* pancreatic ductal/distal bile duct / ampullary / duodenal adenocarcinoma, *INT* intestinal, *NON-INT* non-intestinal, *CK* cytokeratin, *CI* confidence interval.

### Baseline data and biology of the intestinal subtype

Results of a subgroup analysis of INT adenocarcinomas are shown in Table 3. While median tumor size did not differ between INT and NON-INT type (23 vs 25 mm, p = 0.779), several attributes of lower malignant potential could be demonstrated for INT adenocarcinomas. High tumor grade, high T and N Stage, lymph node ratio (LNR), perineural and vascular invasion were significantly less frequent and expression of intestinal molecular markers CK20 and CDX2 was significantly higher with INT versus non-INT adenocarcinoma. INT adenocarcinomas were more frequently located in the ampulla of Vater or duodenum region than in the pancreas or distal bile duct and margin-free surgical resection was more frequently achieved. There was a statistically significant association of the INT type with adenomatous precursor lesions, but not with IPMN precursor lesions.

**Table 3:** Baseline data and biology of the intestinal subtype

**Parameter**		**INT**	**NON-INT**	**p**
**N**		**39**	**159**	**-**
Male : female		25:14	77:82	0.079
Age		69 (36–81)	67 (30–89)	0.569
Distant metastasis (M1)		1 (3%)	6 (4%)	0.714
Neoadjuvant therapy		1 (3%)	13 (8%)	0.220
Resection	PPPD	31 (80%)	132 (83%)	0.701
	Whipple	6 (15%)	17 (11%)	
	TPE	2 (5%)	10 (6%)	
	PVR	8 (21%)	54 (34%)	0.105
Perioperative mortality		2 (5%)	5 (3%)	0.548
Tumor size (mm)		23 (1–320)	25 (3–70)	0.779
Grade G3/4		6 (15%)	66 (42%)	**0.002**
pT Stage 3/4		21 (54%)	134 (84%)	**0.000**
pN Stage 1/2		16 (41%)	116 (73%)	**0.000**
Lymph node ratio (LNR)		.00 (.00-.58)	.12 (.00-.86)	**0.018**
Lymphangiosis (L1)		12 (31%)	75 (47%)	0.064
Hemangiosis (V1)		1 (3%)	28 (18%)	**0.017**
Perineural invasion (Pn1)		13 (33%)	100 (63%)	**0.001**
Positive resection margin		3 (8%)	48 (30%)	**0.004**
Location	PDAC / DBDAC	16 (41%)	133 (84%)	**0.000**
	AMPAC / DUOAC	23 (59%)	26 (16%)	
% CK7		85 (0–100)	90 (0–100)	0.070
% CK20		45 (0–100)	0 (0–100)	**0.000**
% nuclear CDX2		80 (0–100)	0 (0–100)	**0.000**
Associated precursor lesion	Adenoma	8 (21%)	12 (8%)	**0.016**
	IPMN	3 (8%)	8 (5%)	0.516

Values are depicted as absolute and percentage of column for categorial and median (range) for scale variables, p value derived from Mann–Whitney (scale variables) or Chi squared test (categorial variables) for the respective row.

Abbreviations: Whipple / PPPD non / pylorus-preserving pancreatoduodenectomy, *TPE* total pancreatectomy, *PVR* portal venous resection, *PDAC/DBDAC/AMPAC/DUOAC* pancreatic ductal/distal bile duct/ampullary/duodenal adenocarcinoma, *NON-/INT* non-/intestinal subtype, *CK* cytokeratin.

### Survival analysis for tumor location and histological subtype

**Figure 3:**
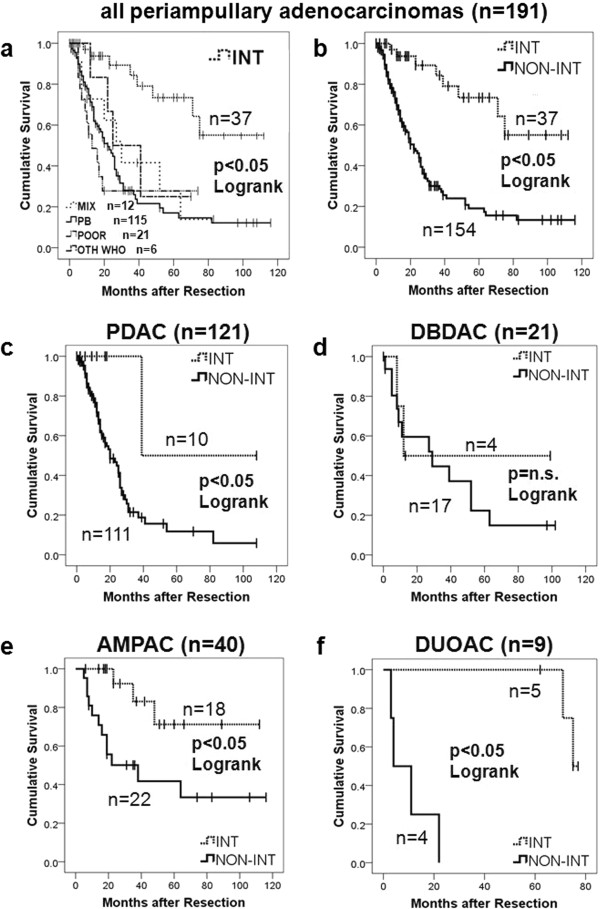
**Survival plots according to histological subtypes and tumor origin. a**: KM plot for all patients with periampullary adenocarcinomas, stratified for histological subtypes. **b**: KM plot for all patients with periampullary adenocarcinoma, stratified for INT versus non-INT subtype. **c-f**: KM plots for INT versus non-INT histological subtype divided by respective periampullary adenocarcinoma origin (pancreatic head (PDAC), ampullary (AMPAC), distal bile duct (DBDAC), duodenal (DUOAC). perioperative deaths (n = 7) excluded, p values derived from two-sided Logrank test. For details see also Table 4. Abbreviations: KM Kaplan Meier, INT intestinal type adenocarcinoma, PB pancreatobiliary type adenocarcinoma, MIX mixed type adenocarcinoma, POOR poorly differentiated carcinoma, OTH other WHO types. n.s. not significant.

Details of survival analysis regarding tumor location and subtype are shown in Table 4 and Figure 3. Perioperative deaths (n = 7) were excluded from survival analysis. Overall median follow-up was 15 months (range 0–116 months). Median survival ranged in ascending order from 23, 29, 64 to 71 months for PDAC, DBDAC, AMPAC to DUOAC. For histological subtypes, median survival was 13, 22, 25 and 30 months for POOR, PB, OTH and MIX subtype, while median survival was not yet reached for the INT subtype. Pairwise comparison by two-sided logrank test among histological subtypes disclosed that significant differences existed only between INT type versus other types. Therefore, non-intestinal types (NON-INT) were assigned to a common group. When survival plots were stratified by tumor location, survival after resection of INT type adenocarcinomas was remarkably favorable for each location, reaching the significance level for PDAC, AMPAC and DUOAC (Table 4 and Figure 3).

**Table 4:** Univariate survival analysis for tumor location and histopathological subtypes in periampullary adenocarcinomas

**Location**	**Type**	**N**	**Events**	**Survival (months)**		**p**
			**(Deaths)**	**Median**	**Mean**	
		**Comparison of tumor locations versus PDAC**				
PDAC	ALL	121	64	23	31	**-**
DBDAC		21	14	29	39	0.430
AMPAC		40	14	64	72	**0.000**
DUOAC		9	6	71	46	0.169
		**Comparison of tumor subtypes versus INT**				
ALL	INT	37	8	NR	83	**-**
	MIX	12	8	30	37	**0.002**
	OTH WHO	6	4	25	38	**0.017**
	PB	115	66	22	34	**0.000**
	POOR	21	12	13	28	**0.000**
	NON-INT	154	90	22	35	**0.000**
		**Comparison INT vs NON-INT stratified for tumor location**				
PDAC	NON-INT	111	63	20	29	**0.034**
	INT	10	1	39	74	
DBDAC	NON-INT	17	12	12	37	0.563
	INT	4	2	29	55	
AMPAC	NON-INT	22	11	38	56	**0.019**
	INT	18	3	NR	90	
DUOAC	NON-INT	4	4	4	10	**0.003**
	INT	5	2	75	75	

Survival estimates are derived from Kaplan-Meier method, p values from two-sided Logrank test. For Kaplan-Meier plots see Figure 3. Perioperative deaths (n = 7) were excluded from survival analysis.

Abbreviations: *INT* intestinal, *NON-INT* non-intestinal, *PB* pancreatobiliary subtype, *MIX* mixed subtype, *POOR* poorly differentiated carcinoma, *OTH* other subtypes, *PDAC* pancreatic ductal adenocarcinoma, *DBDAC* distal bile duct adenocarcinoma, *AMPAC* ampullary adenocarcinoma, *DUOAC* duodenal adenocarcinoma, *NR* not reached.

### Univariate and multivariate survival analysis

Results of uni- and multivariate survival analysis are shown in Table 5. Cases with perioperative death (n = 7) were excluded from survival analysis. In univariate analysis, several clinicopathologic parameters were found to be significantly associated with poor survival: necessity of portal venous resection, high tumor grade, T stage, N stage and LNR, lymphatic and blood vessel invasionas well as positive resection margins. Presence of a precursor lesion was significantly associated with better survival. This was however only the cause for adenomatous precursor lesions in non-pancreatic adenocarcinomas (p = 0.017, two-sided Logrank test), as the subgroup of PDAC with associated IPMN did not show significantly better survival than PDAC without associated IPMN (p = 0.538, Logrank test). Both tumor location and histological subtype were highly significant predictors of survival (p < 0.001). Accordingly, high CK7 expression and low CDX2 expression, as characteristic of NON-INT phenotype, were significant predictors of poor survival.

**Table 5:** Univariate and multivariate survival analysis for resected periampullary adenocarcinomas

**Parameter**	**Condition**	**Median survival (months)**		**Hazard ratio**	**Univariate**	**Multivariate**
		**With condition**	**Without condition**	**(95% CI)**	**p**	**p**
All patients (n = 191)		-26-		-		
Gender	Male	30	25	0.953 (0.641–1.417)	0.812	NI
Age (years)^1^	> 67	26	27	1.050 (0.706–1.561)	0.811	NI
Distant metastasis	Yes	11	27	2.044 (0.892–4.688)	0.091	NI
Neoadjuvant tx	Yes	25	26	1.536 (0.741–3.182)	0.248	NI
PVR	Yes	25	27	1.640 (1.068–2.517)	0.024	0.510
Tumor size (mm)^1^	> 25	26	26	1.069 (0.699–1.635)	0.759	NI
Tumor grade	G3/4	17	31	1.808 (1.199–2.726)	**0.005**	0.075
pT stage	pT3/4	23	41	1.938 (1.160–3.238)	**0.012**	0.905
pN stage	pN1/2	22	52	1.883 (1.198–2.959)	**0.006**	0.211
LNR^1^	> 0.09	20	48	2.291 (1.515–3.465)	**0.000**	**0.019**
Lymphangiosis	Present	22	30	1.557 (1.035–2.342)	**0.033**	0.398
Hemangiosis	Present	12	29	1.900 (1.161–3.109)	**0.011**	0.384
Perineural invasion	Present	25	29	1.322 (0.881–1.984)	0.177	NI
Precursor lesion	Present	NR	25	0.362 (0.175–0.748)	**0.006**	0.169
Resection margins	Positive	19	37	2.368 (1.551–3.617)	**0.000**	0.132
Tumor subtype^2^	See Table 4			1.487 (1.253–1.765)	**0.000**	**0.006**
Tumor location^2^	See Table 4			1.471 (1.175–1.843)	**0.001**	0.270
CK7 + (%)^1^	> 90	20	38	1.757 (1.161–2.657)	**0.008**	0.869
CK20 + (%)^1^	> 0	29	23	0.311 (0.538–1.218)	0.311	NI
CDX2 + (%)^1^	> 10	48	20	0.562 (0.368–0.858)	**0.008**	0.767

Survival estimates are derived from Kaplan-Meyer method, perioperative deaths (n = 7) were excluded, univariate / multivariate p is derived from two-sided Cox regression in a proportional hazards model.

Abbreviations: *CK* cytokeratin, *CI* confidence interval, + positive. ^1^ cut-off at overall median value, ^2^ defined as ordinal variable according to decreasing median survival as indicated in Table 2, pos positive, tx therapy, *PVR* portal venous resection, *NR* not reached.

Parameters found to be significant in univariate analysis were included in a Cox proportional hazards model including all cases with periampullary adenocarcinomas (n = 191 after exclusion of n = 7 cases of perioperative mortality). In this model, only histological subtype (Hazard Ratio 1.5, p = 0.006) and LNR (Hazard Ratio 2.3, p = 0.019) were found to represent independent predictors of survival. This was confirmed by conditional backward elimination of predictive parameters.

## Discussion

Only one group has so far systematically extended the INT/PB concept to the whole spectrum of periampullary adenocarcinomas [3,9]. We were able to validate and extend these findings in our study. From a plethora of prognostic markers identified in univariate analysis, including UICC stage, tumor size, resection margin status and even immunohistochemical markers, only histologic subtype and lymph node ratio qualified as independent predictors of survival. Hereby the present study confirms that histopathological differentiation, in contrast to tumor location, is an independent prognostic factor. It also confirms that the lymph node ratio is a stronger independent prognostic marker than N stage, which has been shown for PDAC, AMPAC and DBDAC before [10,11].

We were able to demonstrate a significantly better survival of the INT subtype not only for AMPAC but also in the subgroups of DUOAC and PDAC, which has not been demonstrated before due to insufficient case numbers [3,9]. Probably due to its relatively rare occurrence (around 10% of PDAC), INT type PDAC has only recently been recognized [12].

The concept of INT and PB differentiation has recently been extended to IPMN. It has been suggested that INT type IPMN can develop into colloid carcinoma (CAC) [13,14], which is associated with better survival [15,16]. We show that in ampullary and duodenal location, INT phenotype is associated with adenomatous precursor lesions, but this is not the case in PDAC, validating its existence as a separate entity apart from colloid carcinoma or invasive IPMN. In addition, one recent study shows no association of intraductal papillary neoplasms of the bile duct (IPNB) with INT type cholangiocarcinoma [17,18]. It may therefore be suggested that an “intestinal pathway” of carcinogenesis is possible via the INT type of IPMN to colloid carcinoma, and duodenal or ampullary adenoma to DUOAC and AMPAC, but also without papillary precursor lesions to INT type PDAC and DBDAC.

On the other hand, blinded application of the INT/PB classification scheme resulted in assignment of two DUOAC to the PB group. One might speculate that if INT type adenocarcinoma can arise in the pancreas, vice versa PB type adenocarcinoma may arise in rare instances in the duodenum due to common embryologic origin, but further evaluation is currently not possible given the exceeding rarity of DUOAC.

As CK7, CK20 and CDX2 are known to be fairly specific markers for the discrimination of INT and PB subtypes in AMPAC and IPMN [14,19-22], it was surprising that this was not the case for PDAC and DBDAC in our study. This finding is noteworthy as it shows that careful morphological assessment by an experienced pathologist outperforms immunohistochemical markers in this respect.

This study has several limitations that have to be mentioned. First, although data was obtained from a prospectively maintained database, all data was analyzed retrospectively. There was a relatively high number of cases assessed for eligibility from which insufficient amounts of tissue were available for the present study due to use for other studies. A selection bias by exclusion of small tumors with few tissue can thus not totally be ruled out. Furthermore we note a relatively high rate of PDAC in our series compared to other recent studies [2,4] on this subject. This may be explained in several ways: first, selection bias excluding small tumors may favor the inclusion of PDAC which are usually larger than AMPAC; furthermore assignment to tumor location was not performed according to the most recent detailed protocols [2,4] and could only be reassessed in a retrospective manner. Misclassification of origin especially in large tumors cannot totally be ruled out. Nevertheless pathologists and surgeons at our institution since long have been aware of this complex issue and extensive surgical and pathological experience exists. We believe that by primary assessment in a standardized protocol as well as detailed re-assessment in the scope of this study, risk of misclassification was reduced to a minimum.

Another current issue concerns resection margin status in PDAC. Some recent studies demonstrated by extensive resection margin workup that the majority of PDAC resections were in fact margin positive (R1) [23-25]. Margin assessment in our standardized protocol was very detailed but not as extensive as in some of the aforementioned studies, thus a number of R0 might in fact have been R1 resections. Nevertheless our data is validated by the fact that margin status was a prognostic factor. More detailed analysis of this issue was not within the scope of this study but will have to be addressed in future studies.

The most important aspect of pathological diagnostic is clinical decision making on the basis of prognostic factors. With respect to the findings of this study, several aspects warrant consideration.

Based on favorable survival, surgical treatment of metastasis may be indicated in INT type AMPAC, as already suggested by others [26]. Given the fact that tumor location is less relevant than subtype, this concept may be extended to PDAC, DBDAC and DUOAC. Especially since criteria for the rarely performed resection of metastatic PDAC are poorly defined [27], subtype might be a valuable adjunct in decision making.

Furthermore, there is still no consensus regarding the indication and regimen for adjuvant therapy in non-pancreatic periampullary adenocarcinomas [28-33]. In clinical practice, treatment regimen for AMPAC, DUOAC or DBDAC are usually extrapolated from PDAC. Limited data suggests that patients with node positive and margin-negative AMPAC benefit from adjuvant therapy [28,30,33]. However, these studies did not report on subtype. Regarding the results of our study, a more differentiated approach based on subtype may be suggested for future trials.

It has been shown that survival figures of series reporting outcome of resected pancreatic head cancer have been biased by inclusion of ampullary cancers [23,34]. In consequence, more thorough examination of tumor origin has been demanded by some authors [2]. However given the results of our study, the biologically valid and logistically preferable approach would be to distinguish between INT and PB differentiation rather than tumor location. This is emphasized by the fact that for larger tumors with involvement of all periampullary structures, assessment of primary location is very difficult or impossible.

## Conclusion

In summary, our study demonstrates that histopathological subtype and lymph node ratio are the most important prognostic factors in periampullary adenocarcinomas, outperforming all other parameters. As tumor location becomes irrelevant with correct microscopical phenotype classification, this may obviate the need for time-intensive macroscopical and microscopical workup of tumor location in pancreatoduodenectomy specimen. It must be recommended that studies and trials on outcome and therapy of periampullary adenocarcinomas stratify for intestinal and pancreatobiliary differentiation.

## Competing interests

The authors have no competing interests to declare.

## Authors’ contributions

PB, IK and MW performed the pathological assessment and critically revised the manuscript. DB, SK and JH collected clinical data and performed the operations. TK and UTH performed the operations and critically revised the manuscript. UFW and FM collected the data, performed statistical analysis and drafted the manuscript. All authors read and approved the final manuscript.

## Pre-publication history

The pre-publication history for this paper can be accessed here:

http://www.biomedcentral.com/1471-2407/13/428/prepub
